# The plasma metabolome of long COVID patients two years after infection

**DOI:** 10.1038/s41598-023-39049-x

**Published:** 2023-08-01

**Authors:** Yamilé López-Hernández, Joel Monárrez-Espino, David Alejandro García López, Jiamin Zheng, Juan Carlos Borrego, Claudia Torres-Calzada, José Pedro Elizalde-Díaz, Rupasri Mandal, Mark Berjanskii, Eduardo Martínez-Martínez, Jesús Adrián López, David S. Wishart

**Affiliations:** 1 CONAHCyT-Metabolomics and Proteomics Laboratory, Academic Unit of Biological Sciences, Autonomous University of Zacatecas, 98000 Zacatecas, Mexico; 2 Department of Health Research, Christus Muguerza del Parque Hospital – University of Monterrey, 31125 Chihuahua, Mexico; 3 Academic Unit of Biological Sciences, Autonomous University of Zacatecas, 98000 Zacatecas, Mexico; 4 The Metabolomics Innovation Centre, University of Alberta, Edmonton, AB T6G 1C9 Canada; 5 Departamento de Epidemiología, Hospital General de Zona #1 “Emilio Varela Luján”, Instituto Mexicano del Seguro Social, Zacatecas, 98000 México; 6 Department of Biological Sciences, University of Alberta, Edmonton, AB T6G 1C9 Canada; 7 Laboratory of Cell Communication & Extracellular Vesicles, Division of Basic Science, Instituto Nacional de Medicina Genómica, 14610 Ciudad de México, Mexico; 8 MicroRNAs and Cancer Laboratory, Academic Unit of Biological Sciences, Autonomous University of Zacatecas, 98000 Zacatecas, Mexico

**Keywords:** Biomarkers, Diseases, Molecular medicine

## Abstract

One of the major challenges currently faced by global health systems is the prolonged COVID-19 syndrome (also known as “long COVID”) which has emerged as a consequence of the SARS-CoV-2 epidemic. It is estimated that at least 30% of patients who have had COVID-19 will develop long COVID. In this study, our goal was to assess the plasma metabolome in a total of 100 samples collected from healthy controls, COVID-19 patients, and long COVID patients recruited in Mexico between 2020 and 2022. A targeted metabolomics approach using a combination of LC–MS/MS and FIA MS/MS was performed to quantify 108 metabolites. IL-17 and leptin were measured in long COVID patients by immunoenzymatic assay. The comparison of paired COVID-19/long COVID-19 samples revealed 53 metabolites that were statistically different. Compared to controls, 27 metabolites remained dysregulated even after two years. Post-COVID-19 patients displayed a heterogeneous metabolic profile. Lactic acid, lactate/pyruvate ratio, ornithine/citrulline ratio, and arginine were identified as the most relevant metabolites for distinguishing patients with more complicated long COVID evolution. Additionally, IL-17 levels were significantly increased in these patients. Mitochondrial dysfunction, redox state imbalance, impaired energy metabolism, and chronic immune dysregulation are likely to be the main hallmarks of long COVID even two years after acute COVID-19 infection.

## Introduction

Historically, highly pathogenic beta-coronaviruses have been associated with severe respiratory diseases. According to the WHO, the severe acute respiratory syndrome coronavirus (SARS-CoV), and the Middle East respiratory syndrome coronavirus (MERS-CoV) were responsible for epidemics in 2002–2003 and 2015, respectively. During the SARS-CoV epidemic, the virus was reported in 29 countries with 8,437 cases and 813 fatalities^1^. On the other hand, MERS-CoV was reported in 27 countries with 2,519 laboratory-confirmed cases between 2012 and 2020, resulting in 866 deaths^2^. In 2019, exactly 100 years after the last pandemic caused by an H1N1 influenza A virus (the Spanish flu), a new pandemic affected almost every country around the world. As of February 26, 2023, over 758 million confirmed cases of SARS-CoV-2 and over 6.8 million deaths have been reported globally. To date, around 653 million patients have recovered^3^. However, as early as spring 2020, people began describing their experiences of not fully recovering from SARS-CoV-2 infection^4^. This extended version of the disease has been called “long COVID”. Interestingly, the term long COVID is a patient-created term promoted in Twitter by Elsa Perego, an archeologist at University College London.

It has been widely described that some viruses lead to persistent physiological alterations even a decade after infection. The term “post-viral syndrome” has been in use for over a century^5^. Chronic symptoms such as fatigue, joint pain, and cardiovascular problems have been reported after recovery from other infections such as the West Nile, Polio, Dengue, Zika, seasonal flu, Epstein-Barr, Ebola, MERS, and SARS^6,7^. However, none of these viruses have affected so many people in the same time window as SARS-CoV-2, which offers the scientific community a unique opportunity to understand the etiology of post-viral syndromes such as long COVID.

Long COVID (also known as post-COVID-19 syndrome or post-acute sequelae of COVID-19 (PACS)) is a condition characterized by long-term or persistent health problems appearing after the initial recovery from COVID-19 infection. The WHO has described long COVID as a condition “that occurs in individuals with a previous history of probable or confirmed SARS-CoV-2 infection, usually three months after the onset, with symptoms lasting at least two months that cannot be explained by an alternative diagnosis”^8^. It is estimated that 30–60% of recovered patients, even after a mild disease, will experience long COVID or symptoms persistence with varying durations^9^. Based on a conservative estimated incidence, at least 65 million individuals worldwide could be experiencing long COVID^3^.

Similar to COVID-19, long COVID affects multiple organ systems, including the respiratory, cardiovascular, nervous, and gastrointestinal systems. More than 50 symptoms have been reported associated with long COVID^10^. Observational studies have reported that some symptomatic conditions are resolved within three months of hospitalization in 50% of patients^11^, but the rate of full recovery drops to 35% between three and 6 months after hospitalization, and to 15% between 6 and 9 months. Importantly, a high proportion of that population has residual lung tissue injury, with detectable radiological abnormalities on chest computed tomography (CT) scans^12^. Fatigue, loss of concentration, headaches, shortness of breath, anosmia, muscle weakness, joint pain are some of the symptoms most reported. Therefore, more than a homogeneous entity, long COVID could be considered as a spectrum of disorders, that affects individuals with complications directly linked to the virus (long-term residual damage in the lungs, brain, or heart), and individuals manifesting systemic unspecific signs/symptoms (fatigue, headache, and arthromyalgias)^13^. The increasing number of patients with long COVID poses a challenge for public health systems around the world, but, currently, there are no guidelines for accurately diagnosing patients with long COVID and classification is still underestimated and subjective.

Both untargeted and targeted metabolomics have proven to be valuable tools for studying of long COVID. Our results, based on an untargeted lipidomics approach,^14^ demonstrate that several species of phosphatidylcholines and sphingomyelins were expressed at higher levels in long COVID-19 patients compared with controls. The paired analysis, which compares patients with an active infection and two years after recovery, showed 170 dysregulated lipidic features.

In the present work we used quantitative targeted metabolomics to evaluate the metabolic reversion of patients with persistent sequelae due to confirmed SARS-CoV-2 infection. Comparison with negative controls allowed us to identify those metabolites persistently dysregulated after two years of the initial infection. Number, type of symptoms as well as metabolic signatures were different in patients experiencing long COVID (arbitrarily defined by us as long COVID class A and class B patients) and recovered patients (non-long COVID). Besides, IL-17 level was increased in patients with the worst disease evolution (class B patients). To the best of our knowledge, this is the first targeted metabolomics study of long COVID patients conducted beyond twenty months post-infection.

## Results

### Demographic, clinical data and symptoms description

Table 1 shows baseline characteristics of patients enrolled in the study. Age was statistically different between negative (healthy) controls and COVID-19 patients. However, differences were not found in the self-reported comorbidities. Six patients (12.5%) developed mild disease; 37 patients developed (77%) moderate/severe disease while five patients (10.4%) developed critical disease. Six patients (12.5%) were reinfected during 2021 and 2022. All patients were fully vaccinated during the period of 2021–2022. Additional information from class A long COVID patients, class B long COVID patients, and recovered (non-long COVID) is provided in Supplementary Table 1.

**Table 1:** Baseline characteristics of participants in the study.

Variable	Controls (N = 37)	COVID-19 (N = 15)	post-COVID-19 (N = 48)	p-value
Age, median (Q1–Q3)	40.5 (37–53.3)	51 (44.3–59.3)	51.5 (43.5–60.8)	0.0079*^a, b, c^
Male gender, n (%)	17 (44.7)	12 (54.5)	28 (58.3)	0.2226
Smoking, n (%)	4 (10.5)	3 (13.6)	7 (14.6)	0.7231
Diabetes	3 (7.9)	3 (13.6)	7 (14.6)	0.7833
Hypertension	9 (23.7)	6 (27.3)	20 (41.7)	0.1035
Obesity	3 (7.9)	0 (0)	5 (10.4)	0.3823
Hemoglobin (g/dL)	15.3 (14.4–16.2)	14.4 (12.6–16.2)	15.4 (14.5–16.1)	0.7833
Platelets (thousands/ mL)	276 (236.5–325.5)	177 (68.2–281.8)	242.5 (219.8–290.5)	0.1035
Leukocytes (× 10^3^)	7.1 (6.0–8.3)	6.7 (4.4–11.1)	7.0 (6.2–7.8)	0.3823
Lymphocyte counts (%)	31.8 (25.5–36.4)	12.1 (4.9–22.1)	33.8 (29–38.9)	< 0.0001*^a,d^
Creatinine (mg/dL)	0.9 (0.7–1.1)	0.7 (0.6–0.9)	0.8 (0.7–1.0)	0.2452

a: negative controls vs. COVID-19; b: COVID-19 vs. post-COVID-19; c: negative controls vs. post-COVID-19; d: COVID-19 vs. post-COVID-19

**Figure 1:**
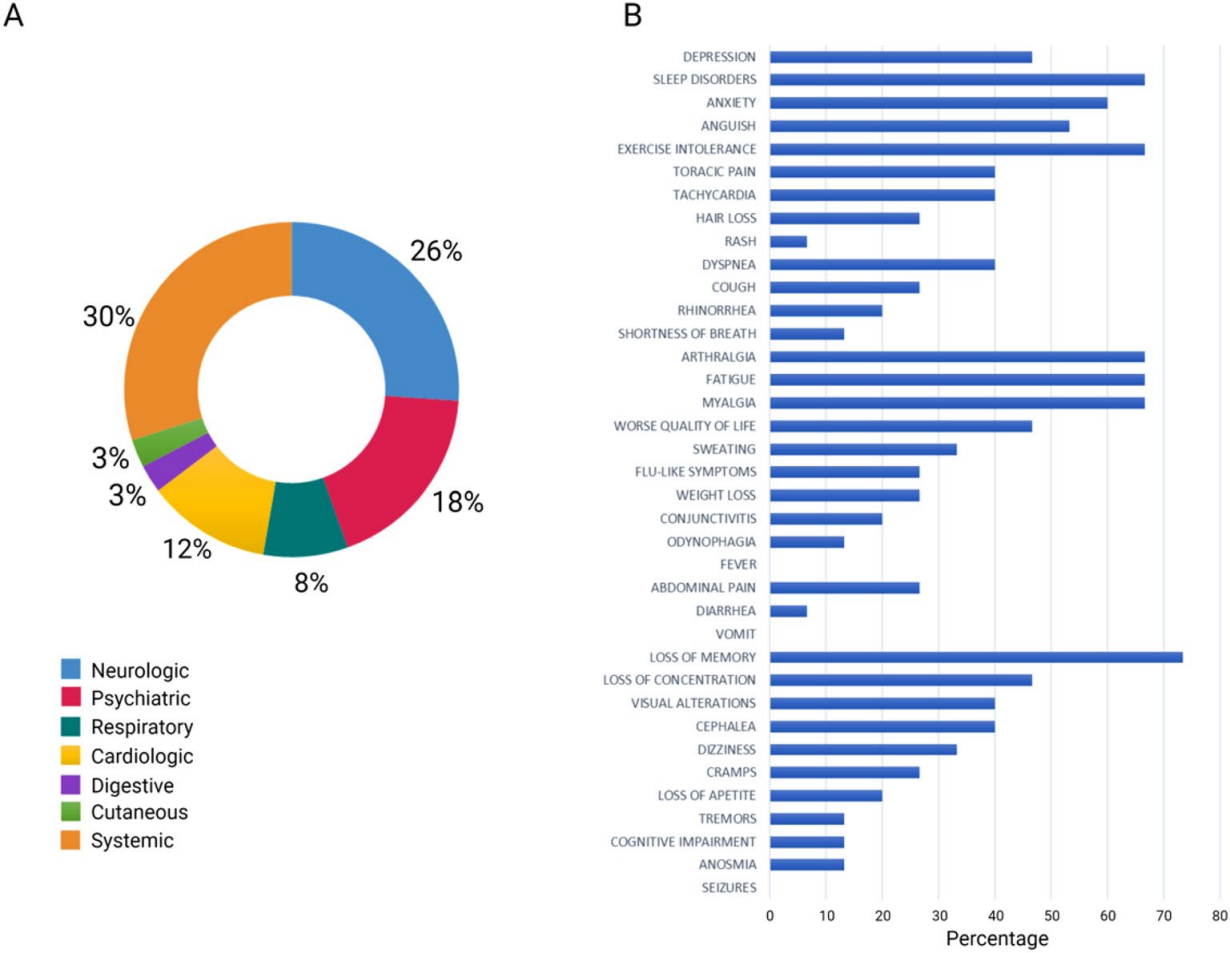
Most common symptoms remaining after 24 months in 48 post-COVID-19 patients. (**a**) Distribution of symptoms according to organ systems. (**b**) Results obtained via in-person questionnaire.

The questionnaire answered by the patients revealed the most persistent symptoms which were grouped into five broad categories: systemic, neurologic, psychiatric, cardiologic, and respiratory. The most predominant symptoms were loss of memory (73.3%), sleep disorders, arthralgia, fatigue, exercise intolerance, myalgia (66.7%), and anxiety (60.0%) (Fig. 1). Class A patients experienced mainly neuropsychiatric symptoms with a co-occurrence of five symptoms and less, while class B patients experienced both neuropsychiatric and systemic symptoms, with a frequency of more than five concomitant symptoms (Supplementary Fig. 1).

### Paired analysis: COVID-19/ long COVID-19 phases

**Figure 2:**
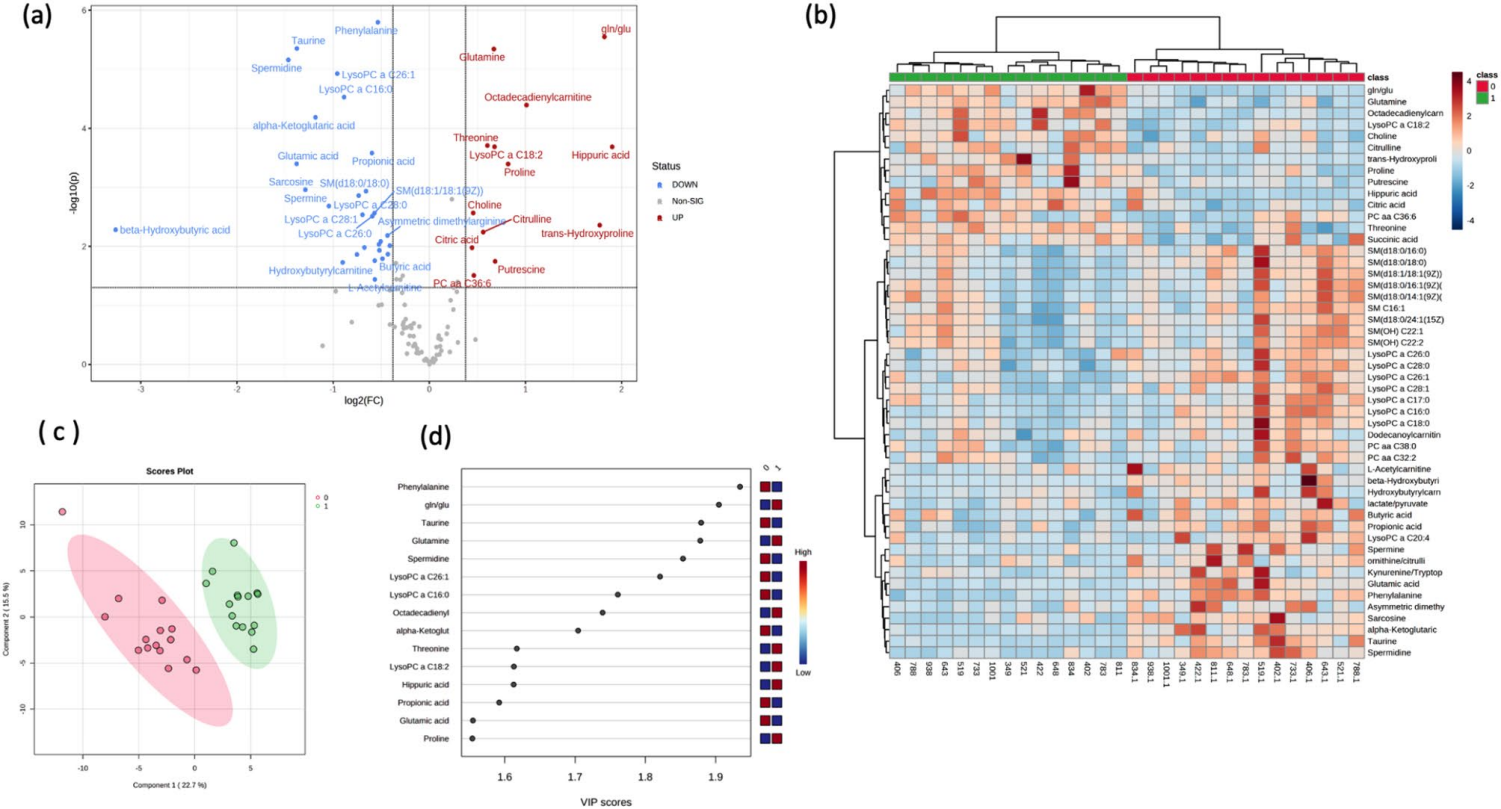
Multivariate analysis. (**a**) The volcano plot of the plasma metabolomics between the COVID-19 phase and the long COVID-19 phase. Fold change threshold = 1.3 and q-value = 0.05 (FDR adjusted). Metabolites highlighted in red are overexpressed in long COVID phase. (**b**) Representative heatmap of top 50 significant metabolites (t-test) in the comparison of COVID-19 and long COVID-19 phases (red (0): COVID-19 phase; green (1): long COVID-19 phase). (**c**) Score scatter plot based on the two-dimensional PLS-DA (red (0): COVID-19 phase; green (1): long COVID-19 phase). (**d**) Rank of the different metabolites (the top 15) identified by the PLS-DA according to the VIP score on the x-axis. The most discriminating metabolites are shown in descending order of their coefficient scores. The color boxes indicate whether metabolite concentration is increased (red) or decreased (blue).

When paired samples (COVID-19/long COVID-19) from 15 patients were compared metabolically, 53 plasma metabolites were found to be significantly different (FDR < 0.05). The volcano plot (Fig. 2a) shows that 13 metabolites were significantly upregulated in the long COVID-19 phase, while 32 were downregulated with a fold change (FC) threshold > 1.3 (FDR < 0.05). Heatmap analysis (Fig. 2b) shows a clustering of patients corresponding to their COVID-19 and long COVID phases, revealing that lysoPCs (except lysoPC 18:2) and SMs were downregulated in the long COVID phase. Multivariate analysis (via PLS-DA) demonstrated a clear separation between both COVID phases (accuracy: 0.97, R^2^: 0.94, Q^2^: 0.77) (Fig. 2c). The VIP plot (Fig. 2d) shows that phenylalanine, taurine, glutamine, and spermidine had lower plasma concentrations in the long COVID-19 phase, while the glutamine/glutamate ratio was increased in the long COVID-19 phase.

### Long COVID-19 patients compared with controls

**Figure 3:**
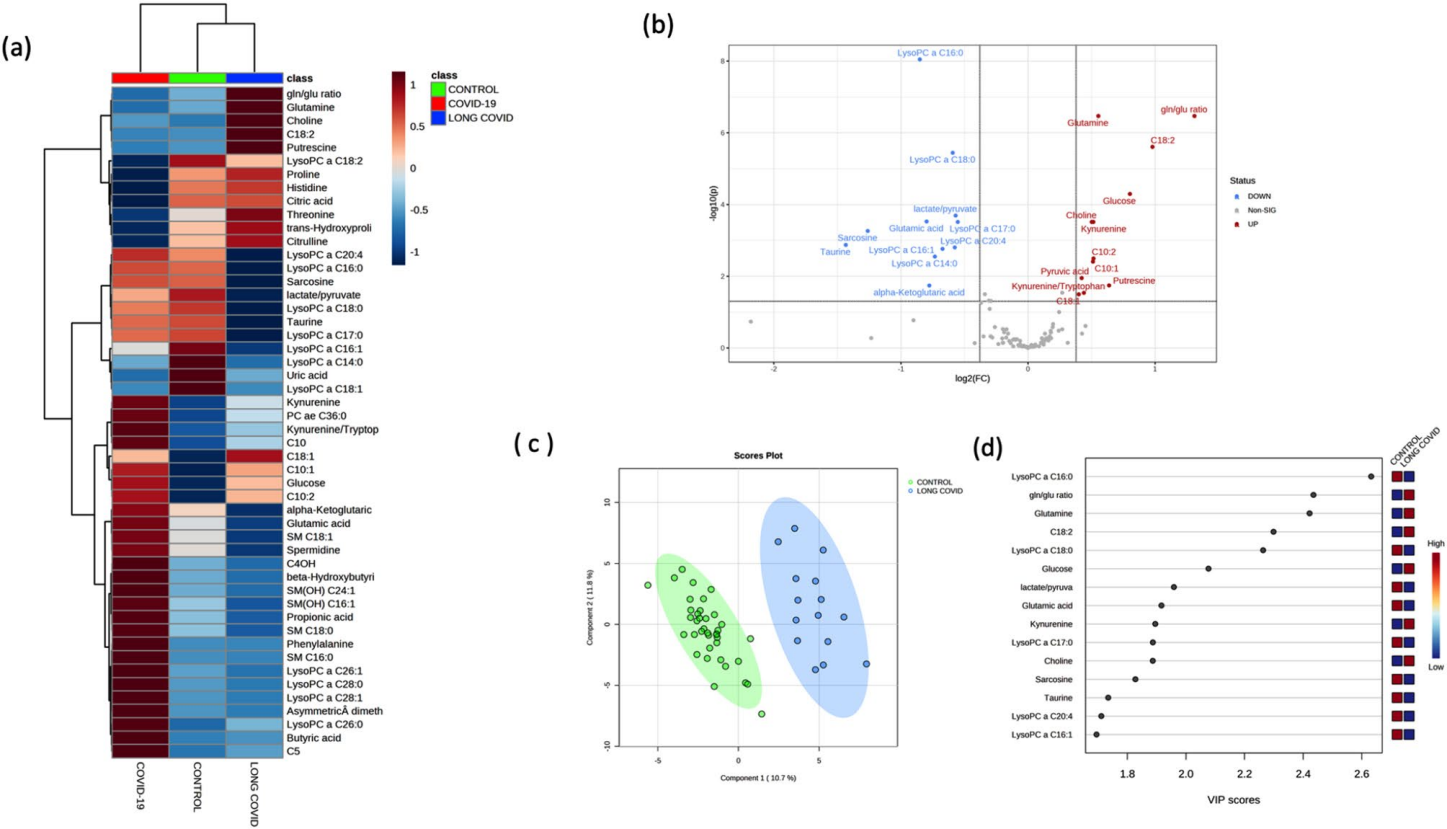
Multivariate analysis. (**a**) Representative average heatmap of top 50 significant metabolites (ANOVA) in the comparison of controls, COVID-19 and long COVID-19 patients. (**b**) The volcano plot of the plasma metabolomics between controls and long COVID-19 patients (red represents the up-regulated metabolites compared with controls, blue represents the down-regulated metabolites compared with controls, and gray represents the metabolites with no difference between both groups). Fold change threshold = 1.3 and p-value = 0.05 (FDR adjusted). (**c**) Score scatter plot based on the two-dimensional PLS-DA. (**d**) Rank of the different metabolites (the top 15) identified by the PLS-DA according to the VIP score on the x-axis. The most discriminating metabolites are shown in descending order of their coefficient scores. The color boxes indicate whether metabolite concentration is increased (red) or decreased (blue).

In order to know if the altered metabolites (and those that remained unsignificant) were dysregulated with respect to normal values, a group of negative SARS-CoV-2 controls (i.e., healthy controls) collected from 2020 was added to the analysis. The heatmap (Fig. 3a) shows the metabolites with significant differences across the three study groups (one way ANOVA). Volcano plots (Fig. 3b) were used to identify the dysregulated metabolites in the long COVID-19 patients with respect to the healthy controls. Two-sample t-tests & Wilcoxon rank-sum tests shows that in comparison with the healthy controls, 27 metabolites were still dysregulated in long COVID patients. C18:2 (adjusted p = 2.4 × 10^–6^), C10:2 (adjusted p = 3 × 10^–3^), C10:1(adjusted p = 3 × 10^–3^), as well as glutamine (adjusted p = 3.3 × 10^–7^), choline (adjusted p = 3 × 10^–4^), glucose (adjusted p = 5 × 10^–5^), kynurenine (adjusted p = 3 × 10^–4^), pyruvic acid (adjusted p = 1.0 × 10^–2^), kynurenine/tryptophan ratio (adjusted p = 2.0 × 10^–02^), putrescine (adjusted p = 0.01), were found in higher concentrations in long COVID-19 patients relative to the healthy controls.

In addition, lysoPC 14:0 (adjusted p = 2.8 × 10^–3^), lysoPC 16:0 (adjusted p = 8.9 × 10^–9^), lysoPC 16:1(adjusted p = 1.7 × 10^–3^), lysoPC 18:0 (adjusted p = 3.6 × 10^–6^), LysoPC 17:0 (adjusted p = 3.0 × 10^–4^), LysoPC 20:4 (adjusted p = 1.5 × 10^–3^), SM(OH)22:1(adjusted p = 0.04), PC aa 32:2 (adjusted p = 0.03), sarcosine (adjusted p = 5.4 × 10^–4^), taurine (adjusted p = 1.3 × 10^–3^), and glutamic acid (adjusted p = 2.9 × 10^–4^) were found downregulated in long COVID-19 patients relative to the healthy controls. Likewise, the glutamine/glutamate ratio (adjusted p = 3.3 × 10^–7^) was increased in long COVID-19 patients, while the lactate/pyruvate ratio (adjusted p = 2.9 × 10^–4^) was found to be decreased.

Glucose, C10:2, C18:1, C10:1, lysoPC 14:0, lysoPC 16:1, lysoPC 18:1, PC ae 36:0, uric acid, C10, and pyruvic acid were found to be in similar concentration levels as for those in the COVID-19 phase.

Several other metabolites previously related with severity in COVID-19 tend towards normal or healthy levels (kynurenine/tryptophan ratio, C18:2, glutamic acid, glutamine, spermidine, kynurenine) in the long COVID-19 group. Of note, a group of sphingomyelins (SM(OH)14:0, SM16:0, SM(18:0), SM(OH)16:1, SM(OH)24:1, SM(18:1), SM(16:1)) were found to be normalized, as well as lysoPC 26:0, lysoPC 26:1, lysoPC 28:1, and lysoPC 28:0, phenylalanine, butyric acid, and propionic acid.

The multivariate analysis (PLS-DA) showed a clear separation between both classes (accuracy: 1; R^2^: 0.98; Q^2^:0.89) (Fig. 3c). The VIP score plot (Fig. 3d) shows that the most important variables that can be used to differentiate negative controls from long COVID-19 patients are phenylalanine, glutamine/glutamate ratio, taurine and glutamine.

### Investigating post-COVID-19 patients: comparison between long COVID class A, class B patients and recovered (non-long COVID)

Differences were found within the post-COVID-19 group, both in the frequency of symptoms reported, and in the plasma levels of some metabolites such as lactic acid, with a bimodal distribution across the group. Therefore, these patients were subclassified according to our own scale as a surrogate for disease severity. 18 patients did not report any symptoms (recovered or non-long COVID). 18 patients reported less than five persistent symptoms (class A long COVID), while 12 reported more than five symptoms (class B long COVID).

We measured the levels of ammonia (in the form of plasmatic urea) in class B patients. The concentration of urea in COVID-19 phase was 43.8 ± 7.35 mg/dL, and 32.8 ± 3.1 mg/dL in long COVID phase. Although the urea levels were lower during the long COVID phase (falling within normal values), no significant differences were found between the two phases (t-test for paired samples, p = 0.146). Blood urea nitrogen (BUN) was similar for all post-COVID patients.

**Figure 4:**
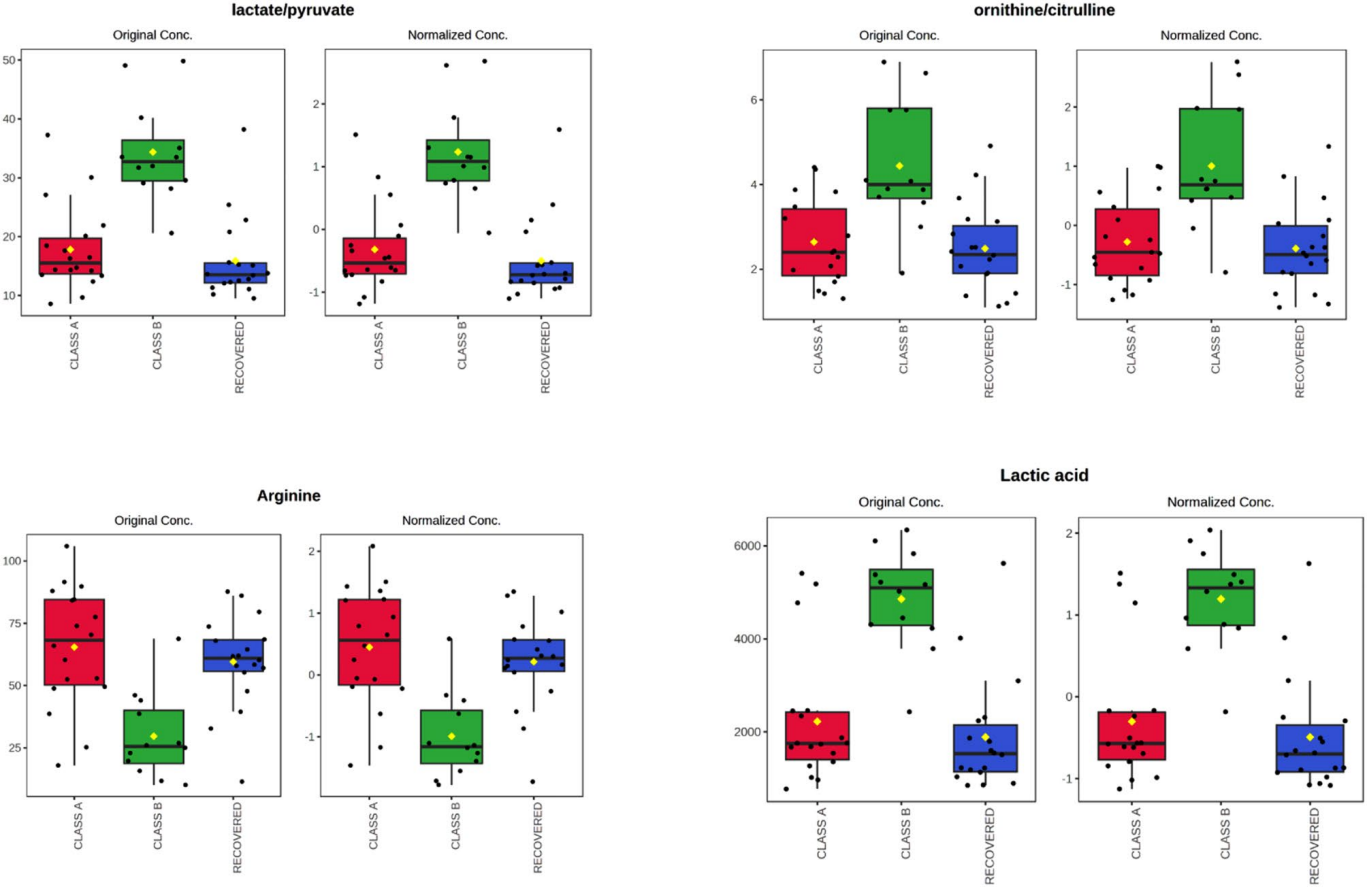
Box plots for some significantly altered metabolites and ratios (p < 0.05) in plasma of class A patients (less than five symptoms), class B patients (more than five symptoms), and recovered patients. The bar plots show the original and normalized values (mean +/− one standard deviation). Medians are indicated by horizontal lines within each box.

Figure 4 shows the box and whisker plots based on one way ANOVA for class A, class B, and fully recovered patients. The lactate/pyruvate ratio (adjusted p value = 5.9 × 10^−6^), lactate (adjusted p value = 1.3 × 10^–5^), arginine (2.0 × 10^–3^), ornithine/citrulline ratio (adjusted p value 2.0 × 10^–3^) were the variables best able to differentiate long COVID patients with more than five symptoms from patients with less than five symptoms. Arginine negatively correlated with the number of symptoms. The differences in glucose levels between class A and class B patients were found to be non-significant (p = 0.09). To evaluate the correlation of symptoms, significant variables, and the laboratory data, an integration of the metadata was done (Supplementary Fig. 2). A negative correlation between arginine and various symptoms, including anxiety (r = − 0.54), fatigue (r = − 0.57), loss of concentration (r = − 0.53), thoracic pain (r = − 0.46), dyspnea (r = − 0.42), myalgia (r = − 0.47), arthralgia (r = − 0.42) as well as with IL-17 (r = − 0.62) was observed.

For differentiating class B long COVID patients from all other post-COVID-19 patients, the lactate/pyruvate ratio had the best performance (AUC: 0.94 (0.85–0.99), sensitivity: 0.92 (0.87–0.97), specificity: 0.94 (0.91–0.98.

### Pathway analysis

**Figure 5:**
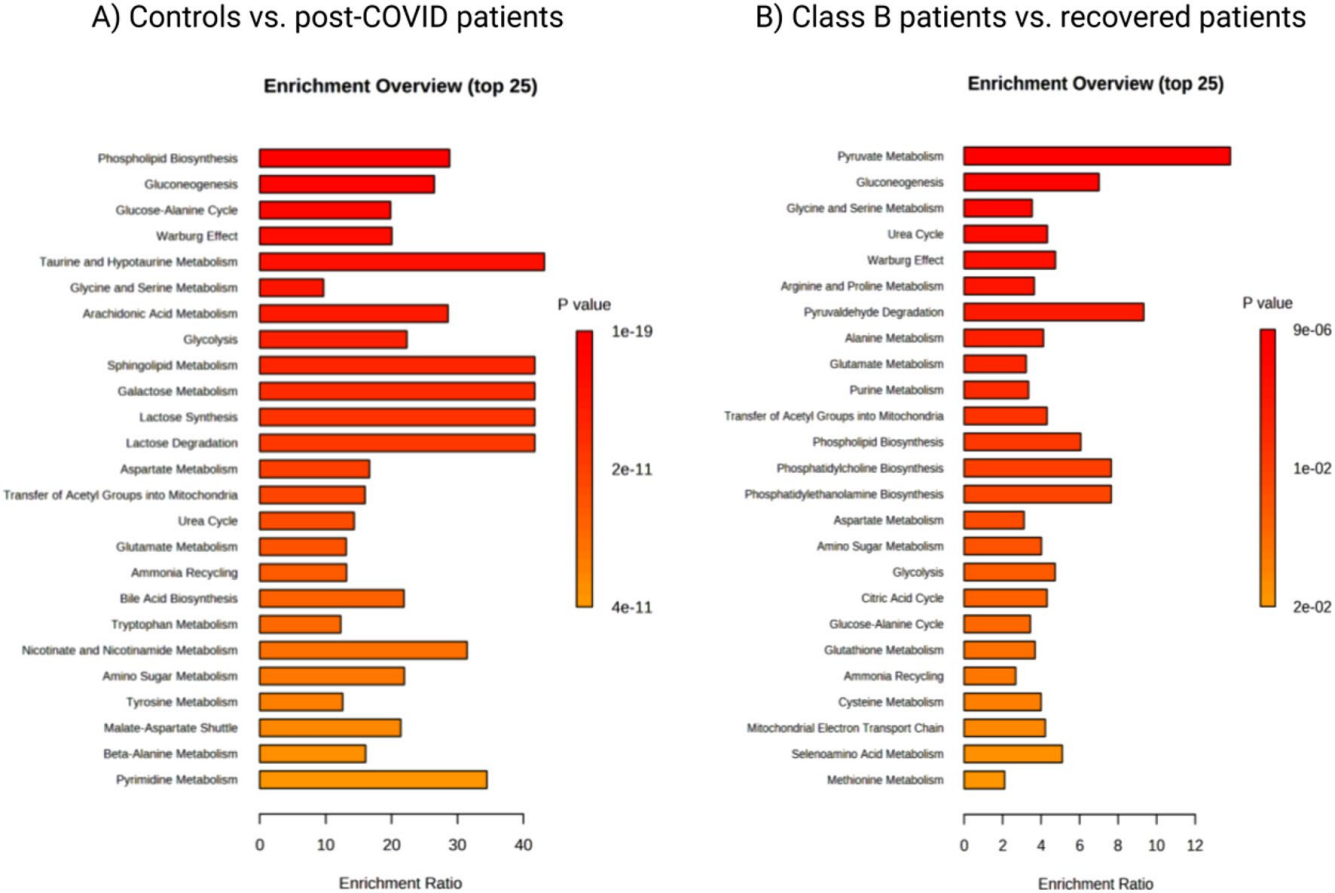
Metabolic pathway analysis. Predicted metabolic pathways with p-value ≤ 0.05 are listed. (**a**) controls vs. post-COVID-19 patients. (**b**) class B patients vs. recovered patients.

Our pathway enrichment analysis (Fig. 5) shows that the top five metabolic pathways significantly dysregulated (FDR < 0.05) in post-COVID patients (relative to controls) were: phospholipids biosynthesis, gluconeogenesis, the glucose-alanine cycle, the Warburg effect, and taurine and hypotaurine metabolism. When comparing class B patients with those recovered, the top five metabolic pathways (FDR < 0.05) were: pyruvate metabolism, gluconeogenesis, glycine and serine metabolism, urea cycle metabolism, and the Warburg effect.

### Plasma IL-17 and leptin

**Figure 6:**
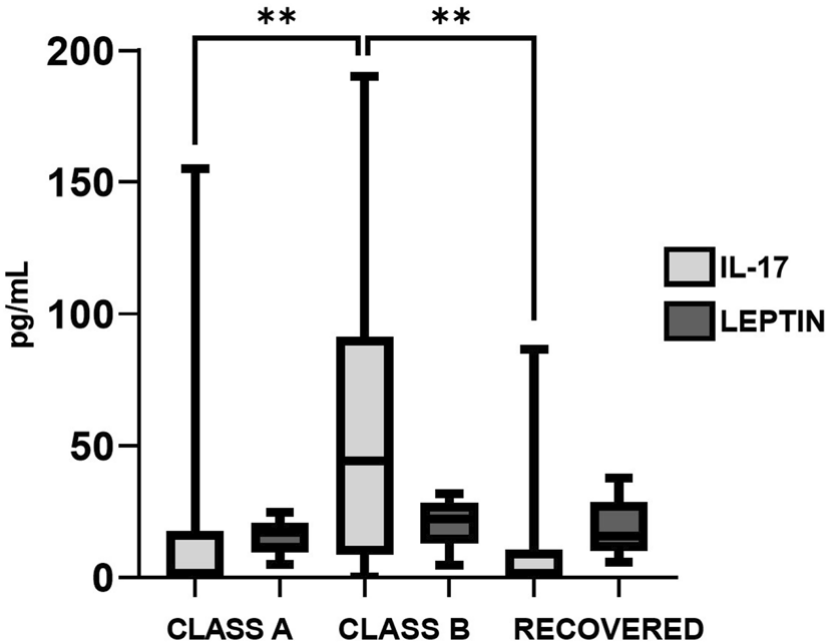
Concentrations of IL-17 and leptin measured by ELISA in post-COVID-19 patients. Graphs were constructed in GraphPad Prism v8.0. The ** p value < 0.01 was calculated using Kruskall-Wallis tests with Dunn´s post-tests.

Figure 6 shows plasma concentrations of IL-17 and leptin as measured by ELISA. IL-17 was significantly increased in class B patients relative to class A patients (Mann–Whitney test, p = 0.0073) and recovered patients (Mann–Whitney test, p = 0.002). Leptin did not show any statistically significant differences in the three-group comparison.

## Discussion

Cumulative evidence from the last three years supports the dysregulation of metabolic and immune markers due to SARS-CoV-2 infection^14^. A retrospective cohort study has demonstrated that COVID-19 patients have a significantly higher risk to develop subsequent autoimmune diseases such as rheumatoid arthritis, ankylosing spondylitis, systemic sclerosis, type I diabetes mellitus, among others^15^. In the present work, our aim was to evaluate the persistence of long-term metabolic alterations in long COVID-19 patients, as well as to measure immune markers that, when chronically produced, can trigger autoimmune diseases.

Since well-defined classification or diagnostic criteria are not available for the long COVID assessment, there is an urgent need for molecular methods able to stratify patients according to the severity of the symptoms they are experiencing. Quantitative and validated scales, such as HAM-A, HAM-D, MoCA and mMRC are considered gold standards for neurocognitive impairment and for dyspnea assessment. However, their practical utility could be limited for complex conditions such as long COVID where a broader range of self-reported symptoms with different severity and duration are present. It has been reported that some long COVID-19 patients complain about extreme cognitive disorders (self-reported symptoms) but without any objective alterations, while others do not report symptoms but exhibit severe cognitive disorders after 6 to 9 months following SARS-CoV-2 infection^16^. In our study, several symptoms were corroborated through objective measures, but with lower rates when using validated scales. Therefore, molecular markers are urgently needed for the correct classification of patients.

Our results revealed that 50% of analyzed plasma metabolites showed statistical differences between COVID-19 and long COVID-19 phases in patients with a more complicated evolution. One of the most dysregulated metabolites was glucose. Montefusco et al.^17^ reported glycemic abnormalities in recovered patients two months after the onset of disease. The hyperglycemic state has been reported to be even worse in hospitalized patients, pointing to a possible causal role of administered drug regimens, including remdesivir and corticosteroids. These drugs stimulate hepatic gluconeogenesis from amino acids released from muscles, which then inhibits glucose uptake^18^. Long COVID has been associated with new-onset insulin resistance which may contribute to the onset of depressive symptoms by enhancing overall neurotoxicity^19^.

A number of other metabolites were also found to be dysregulated. Increased plasma pyruvate levels could be both a consequence of glycolytic dysregulation and protein degradation. The increase in putrescine levels in the long COVID phase may be an indicator of increased protein degradation to help fuel pyruvate metabolism.

Taurine and spermidine were found significantly decreased in the long COVID phase, although a trend towards normalization was observed when compared with controls. Decreased levels of serum taurine have been observed in patients with Myalgic Encephalomyelitis/Chronic Fatigue Syndrome (ME/CFS)^20^. The depleted levels observed in long COVID-19 phase could explain at least in part the fatigue, since taurine has multiple roles in skeletal muscle, the central nervous system, and energy metabolism. Nevertheless, based on our results, we did not find any correlation between fatigue or myalgia and taurine concentrations in long COVID-19 patients. Holmes et al.^21^ found that taurine levels were increased in post-COVID-19 patients, suggesting hepatic injury, hepatotoxicity, or muscle damage. However, the cohort evaluated in the Holmes study had a three-month follow-up after the initial infection, which is much shorter than the follow-up used in this study.

Furthermore, we observed increased levels of kynurenine, and a trend towards normalization in tryptophan and the kynurenine/tryptophan ratio in long COVID-19 patients. This indicates that, although lower in magnitude, the inflammatory conditions attributable to the hyperactivation of this metabolic pathway are still present and may account for some persistent physiological symptoms in these patients. Increased levels of hippuric acid in the long COVID-19 phase could be associated with a residual intestinal dysbiosis. This metabolite has been found increased in patients with chronic kidney disease and several age-related conditions^22^. An increase in the plasma levels of hippuric acid may also be either the result of an increased fruits and vegetable intake.

Our study revealed increased levels of metabolites associated with collagen metabolism in long COVID patients. Among these metabolites, proline is particularly noteworthy due to its involvement in protein structure and function, as well as its role in maintaining cellular redox homeostasis through the generation of ATP and reactive oxygen species (ROS) during its catabolism. Proline can be synthesized from arginine through various enzymes, including arginase (both type I and type II), ornithine aminotransferase, and P5C reductase^23^. The glutamate/P5C synthase pathway in the intestine is responsible for most of the proline synthesis in the body. The increased levels of proline may arise from arginine or glutamine pathways, potentially in response to hypoxia^24^ or tissue damage. Elevated blood levels of hydroxyproline have been proposed as a biomarker for diseases characterized by fibrosis, indicating an increased demand for proline in collagen synthesis. Trans-hydroxyproline plays a crucial role in collagen synthesis and contributes to the thermodynamic stability of the triple-helical conformation of collagen and associated tissues^25^. In our study, both class A and class B patients had partial lung recovery, as evidenced by persistent lung function alterations observed in the lung CT scans (Supplementary Fig. 3). However, the measured levels of trans-hydroxyproline did not show a significant correlation with these findings. Nonetheless, the potential presence of collagen vascular diseases cannot be ruled out, as they may contribute to a more widespread systemic dysfunction, sometimes associated with viral infections^26^.

The increase in glutamine (and decrease in glutamate levels) indicates a partial reestablishment of critical processes that took place during the COVID-19 infection phase, such as severe immunometabolic dysregulation. During COVID-19 phase, a decrease in circulating levels of glutamine has been widely described^27–32^. This depletion is associated with the consumption of glutamine to generate ATP and precursors (purines and pyrimidines) for the synthesis of macromolecules to assemble progeny viruses; to fuel TCA cycle (as in cancer cell metabolism); to regulate the function of immune cells for maximal cytokine production, lymphocyte function, and for the growth, plasma T cell differentiation, and antibody production by B lymphocytes; and to promote interorgan nitrogen exchange, ammonia detoxification, and pH homeostasis. Glutamine/glutamate pathway is therefore closely related to energy metabolism. Dysregulations in this axis have been previously associated with increased risk of type 2 diabetes and other inflammatory diseases^33,34^. In the post-COVID phase, by the contrary, an increase in glutamine concentrations and a decrease in glutamate (higher glutamine/glutamate ratio) could be associated with long-term recovery, since glutamine demand for immune activation, nucleotide synthesis and ammonia detoxification has decreased. However, in long COVID patients experiencing multiple symptoms (neuropsychiatric and systemic), immune dysregulation persists, leading to a persistent imbalance in glutamine/glutamate metabolism, since glutamine may act as a signaling metabolite. Disruption within the glutamatergic pathway can lead to important neurological consequences, such as cognitive deficits^35,36^. In fact, this exquisitely sensitive glutamine/glutamate homeostasis has been reported disturbed in schizophrenia^37^ and frontotemporal dementia^38^. The glutaminergic dysfunction could be associated with some psychiatric and neurologic symptoms like those reported in the present work.

Alterations in lipid metabolism are evident in most long COVID-19 patients. These patients exhibited significantly higher levels of carnitine and some short, medium, and long acylcarnitines. These alterations have been largely associated with altered fatty acid metabolism, dysfunctional mitochondria-dependent lipid catabolism, and immune processes or the lysis of white blood cells. Similar results have been reported by Guntur et al.^39^, pointing to mitochondrial dysfunction, as was also recognized during COVID-19 acute phase. Besides, decreased levels of LysoPC 16:0, LysoPC 17:0, LysoPC 18:0, and LysoPC 20:4, were found with respect to negative controls. These reductions have been reported in other inflammatory conditions^40^ and other septic processes^41^. Depleted levels of lysophosphatidylcholines and phospholipid ethers, as well as depleted levels of PCs, can impede mitochondrial respiration, as has been also demonstrated in ME/CFS^42^. In line with the lipid dysregulation demonstrated by the targeted metabolomic analysis, routine clinical laboratory tests exhibited elevated levels of total cholesterol, triglycerides, and VLDL, as well as normal levels of HDL and LDL. Xu et al.^43^ found increased LDL, triglycerides, total cholesterol, and decreased HDL in survivors of COVID-19, based on a large observational study with participants from the US Department of Veterans Affairs database compared to controls who had never tested positive for COVID-19.

As positive findings for the metabolic state of long COVID patients, we found that 30 metabolites fell within normal levels. Phenylalanine, which has been widely associated with sepsis and COVID disease severity^14^ decreased to normal levels. Beta-hydroxybutyric acid and citric acid were also normalized, indicating partial recovery of the tricarboxylic acid cycle^14,44^. Butyric acid and propionic acid, two short-chain fatty acids that were found to be altered during COVID-19 phase, also fell within normal levels in post-COVID-19 patients, probably indicating that the leaky gut phenomenon and gut dysbiosis detected during COVID infection could be partially reestablished^45^. Spermidine was also normalized. The decrease in spermidine levels could reflect a trend for normalization in overall redox balance. Although excessive levels of spermidine (as those reported in COVID-19 patients) trigger the production of superoxide radicals, optimal concentrations mitigate oxidative stress and diminish overall ROS production^46^.

In addition, sphingomyelins and long-chain monounsaturated and saturated LysoPCs were found to be within normal levels. We previously noted altered sphingolipids levels during COVID-19 infection^14^. Sphingolipids play a crucial role in the regulation of signal transduction pathways and in certain pathological conditions, such as inflammation-associated illnesses and innate immune response.

In a recent report, Holmes et al.^21^ found a high degree of interindividual variability in follow-up patients, reflecting the heterogeneity of post-COVID-19 patients and the fact that long COVID is a spectrum of disorders. Indeed, computationally modeling of the long COVID phenotype data based on electronic healthcare records found six distinct clusters, each with distinct profiles of phenotypic abnormalities^47^. Since symptom classification is still highly subjective, we decided to arbitrarily classify long COVID patients as: class A (less than five symptoms, mainly neuropsychiatric disorders), and class B (more than five symptoms, with a broad spectrum of systemic disorders). In a recent article^48^, the authors proposed a definition of PASC based on self-reported symptoms, identifying four distinct clusters with concomitant symptoms ranging from two to six, to address the heterogeneity of long COVID. The four PASC subgroups were identified as follows: cluster 1: loss of or change in smell or taste; cluster 2: post-exertional malaise and fatigue; cluster 3: brain fog, post-exertional malaise, and fatigue; cluster 4: fatigue, post-exertional malaise, dizziness, brain fog, gastrointestinal symptoms, and palpitations. Our own classification scale aligns with this study, both in terms of the number and types of symptoms included.

We believe that metabolic information may complement, and partially explain the phenotypic differences among long COVID-19 patients. Xu et al.^49^ classified recovered patients based on abnormal pulmonary functions, finding increased levels of triacylglycerols, phosphatidylcholines, prostaglandin E2, arginine, and decreased levels of betaine and adenosine in patients with abnormal pulmonary function.

In our work, lactic acid levels were increased in patients with more than five symptoms and systemic disorders (class B patients). Ghali et al.^50^ found that patients with ME/CFS exhibited elevated blood lactate at rest. Mitochondrial dysfunction, with increased blood lactate, low levels of ATP, and increased levels of oxidative stress markers have been associated to these alterations^51^, as well as relative deficiency of mitochondria type I fibers on muscle biopsies, and low intracellular pH during recovery phase^52–54^. De Boer et al.^55^ also reported altered lactate levels in long COVID patients, suggesting that long COVID patients have significant impairment in fat beta-oxidation and increased blood lactate accumulation even during low-intensity exercise. In contrast, Guntur et al.^39^ reported low levels of lactic acid and pyruvate in long COVID patients. However, this study was conducted in non-hospitalized patients who had recovered from COVID-19 in March 2020.

Increased level of the lactate/pyruvate ratio in class B patients is another important indicator of mitochondrial dysfunction. The lactate/pyruvate ratio has been proposed as a marker for mitochondrial disorders since it indirectly reflects the NADH/NAD + redox state^56^, lipid metabolism (fat oxidation), and ATP generation. In our study, both markers (lactate and the lactate/pyruvate ratio) were found positively correlated with fatigue, myalgia and arthralgias (Spearman correlation, R > 0.6, p < 0.05).

The increased ornithine/citrulline ratio level in class B patients reflects abnormal metabolic activity in the urea cycle. It is notable that Yamano et al.^57^ reported a similarly increased ornithine/citrulline ratio in CFS patients. An adequate balance of citrulline and ornithine is vital for the clearance of ammonia via urea cycle^58^. If ammonia accumulates intracellularly, the aerobic utilization of pyruvate to feed the TCA cycle is inhibited, resulting in lactate production, which further contributes to fatigue.

In addition, class B patients had decreased levels of arginine in comparison with the other subgroups. The reduced bioavailability of arginine to produce adequate levels of nitric oxide in endothelial cells and vascular tissues leads to the impairment of multiple physiological functions of skeletal muscles, including contractile functions, and muscle repair^59^. Arginine is also a substrate for ornithine production by arginase. It is well known that under certain inflammatory conditions, arginase activity is increased^59^, producing an excess of ornithine and an imbalance in the urea cycle.

Previous studies have pointed to the persistent immune dysregulation following COVID-19 infection^60^. We found increased levels of monocytes in class B patients. Nuber-Champier et al.^61^found that monocyte percentage in the acute phase of the disease allowed them to distinguish between patients with anosognosia for memory deficits in the chronic phase (6–9 months after SARS-CoV-2 infection) and nosognosic patients.

We also measured IL-17 levels in post-COVID-19 patients since it is well known that this cytokine is persistently altered in several chronic inflammatory and autoimmune diseases^62^, and previous reports have indicated an increased risk of such diseases in COVID-19 patients^15^. IL-17 is a proinflammatory cytokine mainly produced by T helper type 17 cells, playing a vital role in the regulation of host immune response against SARS-CoV-2. IL-17-induced dysregulated immune responses have been shown to potentially cause hyperinflammatory COVID-19 disease^63^. It has been reported that IL-17 downregulates protein phosphatase 6, resulting in increased arginase-1 expression in psoriatic keratinocytes^64^. IL-17A has been found to be associated with neurological sequelae and pulmonary fibrosis in post-COVID-19 patients^65,66^. Fluctuations in IL-17 have been associated with fatigue and fatigue severity in ME/CFS patients^67^. Additionally, we measured leptin as it is believed to cause inflammatory fatigue. Leptin is produced mostly by adipose tissue and plays a role in regulating food intake, basal metabolism, and the β-oxidation of fatty acids. In metabolic diseases such as obesity, chronically elevated levels of leptin are observed, which can induce the production of proinflammatory molecules and impair immune self-tolerance, predisposing to develop conditions such as rheumatoid arthritis, inflammatory bowel disease, multiple sclerosis, and others^68^. Increased levels of leptin have been associated with higher fatigue scores in people with CFS^69^. In a study conducted by Stringer et al.^69^, the authors demonstrated that daily fatigue severity was significantly correlated with daily serum leptin levels in women with CFS. However, in our study, we did not find any statistical differences in leptin levels, despite observing higher levels in class B patients. Based on our results, levels of leptin cannot be associated with fatigue, which suggest that the action of IL-17 on metabolic pathways may play a more significant role in this regard.

Metabolomics is not only useful in providing a snapshot of transient physiological or pathophysiological processes taking place in a living organism, but it has also proven to be a powerful tool for proposing and monitoring therapeutic interventions. In the case of long COVID, a common situation worldwide is that patients have reported an absence of adequate support and a poor recognition of their condition, initially attributed to psychiatric issues. People with long COVID have tried a vast range of self-prescribed medicines, supplements, remedies, and dietary changes to manage the disease and to overcome the effects it has on their quality of life and work capacity. Based on our findings, some interventions could be tested for treating long COVID patients: (1) supplementation of taurine (reducing musculoskeletal disorders); (2) supplementation of citrulline (enhancing ammonia clearance and reducing blood lactate, as well as increasing arginine bioavailability for adequate NO production); (3) supplementation of glutamine (primary source for neurotransmitters and immune function balancing); (4) supplementation of antioxidants such as N-acetylcysteine or NAD + (redox balance); (5) supplementation of arginine (targeting endothelial dysfunction in Long-COVID), as has been previously suggested by Tosato et al.^70^. Similarities found in our results with the ME/CFS pathophysiology may pave the way to common therapeutic interventions for both diseases.

We need to acknowledge several limitations with this study. The small sample size was due to the limited number of patients who agreed to participate. While several objective measures of mood and cognition (HAM-A, HAM-D, MoCA mMRC) were used, the sample size did not allow for stratification of patients according to the different test scores obtained, and only self-reported symptoms were used for sub-group classification. Furthermore, we were unable to have a detailed tracking of treatments, medications or alternative therapies during the period evaluated. This limited our interpretation with regard to the impact of pharmacological interventions on the metabolome. Also, some compounds such as hexoses were measured by direct injection (DI); therefore, it was not possible to differentiate glucose (the most abundant sugar) from its epimers. We did not have access to Ct values from the electronic files of the patients indicating the initial viral load. All the patients were infected with the original strain, which was the predominant strain circulating in 2020. There are limited studies examining the association between the initial viral load, as determined by Ct values, and the various long COVID-19 effects^71^. A study conducted in Mexico found that patients with a low Ct experienced between 15 and 20 symptoms, while patients with a high Ct value experienced fewer than six symptoms. Asymptomatic patients with Ct values between 33–36 showed no or very few post-COVID-19 symptoms^72^. However, in larger studies conducted more recently^73^, the focus has shifted towards viral persistence as a potential mechanism for long COVID, rather than solely considering the initial viral load.

Patients from two different hospitals participated in our study: one private hospital located in Chihuahua city, and one public hospital located in Zacatecas city. In general, private hospital patients have relatively high incomes while public hospital patients have lower incomes. Logistic regression models showed no effects of sex, age, comorbidities, vaccination status or severity during the acute phase in the metabolomic profile associated with long COVID. However, patients from the public hospital reported more systemic symptoms in general, while patients from the private hospital reported principally neuropsychiatric symptoms. A recent study found that patients diagnosed with a post-COVID-19 condition were more likely to be unemployed or on public health insurance, illustrating racial and social disparities in access to and experience with healthcare, at least in the USA^74^. Whether the socioeconomic conditions and lifestyles, along with causes of biological origin influence the metabolic phenoreversion of patients recruited in our study, needs to be further investigated. This is particularly important in countries with significant health system disparities and significant differences in population life conditions.

At the moment of this study, most of the negative controls recruited in 2020 were tested positive for COVID-19 in subsequent waves in 2021 or 2022. Therefore, we could not compare the prevalence of symptoms in post-COVID-19 patients with a non-COVID-19 matched group. In a study conducted by Ballering et al.^75^, of the 76 422 participants longitudinally surveyed before and after COVID-19, 4231 (5.5%) had COVID-19 and were compared with 8462 matched controls. The proportion of participants who had at least one core symptom of substantially increased severity to at least moderate was 21.4% in COVID-19 participants versus 8.7% in controls, suggesting that core symptoms were attributed to COVID-19 in 12.7% of participants. These numbers are at present increasing as more epidemiological data are reported worldwide.

## Conclusions

To our knowledge, this study is the first describing quantitative metabolic perturbations two years after the initial acute COVID-19 infection using targeted metabolomics. The evolution of post-COVID-19 patients is different, and symptoms are associated to distinctive metabolic patterns resembling, to some extent, the ME/CSF condition. Moreover, the differences observed between the phenotypes of post-COVID-19 patients reveals potential biomarkers that, once validated in larger and heterogeneous populations, and integrated together with clinical and sociodemographic data, will enable a more accurate and precise classification of long COVID patients beyond classification via self-reported symptoms.

## Methods

### Patient recruitment

For the aims of this study, COVID-19 patient survivors (with confirmed diagnostic based on a positive PCR for SARS-CoV-2) who developed a mild, severe, or critical disease, and were admitted (or hospitalized) in the Instituto Mexicano de Seguridad Social (Zacatecas city, Mexico) and Christus Muguerza del Parque Hospital (Chihuahua city, Mexico) between March and November 2020, were recruited. Participants were contacted for a face-to face interview. They were invited to respond to a questionnaire and to donate a blood sample. Plasma was isolated from the donated blood. COVID-19 patients from the Instituto Mexicano de Seguridad Social were recruited from an initial set of 124 COVID-19 patients enrolled in a previous research study^76^. Of these, 44 (35.6%) passed away during hospitalization and in the following months after hospital discharge. From 80 survivors, it was possible to contact 36 (by their Social Security Number or personal/relative phone number kept in hospital records), and 15 agreed to participate. For these 15 patients, paired plasma samples from the first diagnosis of the acute disease (COVID-19 group) and post-COVID phase were available.

Additionally, from a cohort of patients that were hospitalized in 2020 in Christus Muguerza del Parque Hospital, 33 were randomly selected by age stratification. For those patients, a basal blood sample was not available; however, all clinical information and chest computed tomography (CT) scans were recorded in the hospital archive.

For the neuropsychological assessment, the validated Hamilton Anxiety Rating Scale (HAM-A)^77^ was used. For depression assessment, the Hamilton Depression scale (HAM-D) was used^78^. The Montreal Cognitive Assessment (MoCA) was employed for cognitive impairment^79^. For dyspnea assessment, the modified Medical Research Council (mMRC) dyspnea scale was implemented^80^. Basic blood biochemical markers were performed (i.e., hemoglobin, platelets, leukocytes, lymphocytes, and creatinine) for all enrolled patients.

To assess for differences in the severity of long COVID patients, our own classification was made (arbitrarily) considering the frequency of concomitant symptoms. Recovered patients were classified as those who did not report persistent symptoms. Long COVID was considered if patients reported at least one persistent neurologic, psychiatric, gastrointestinal, cardiologic, respiratory, or systemic symptom. The class A long COVID patients were those reporting less than five persistent symptoms (17 patients), while class B long COVID patients were those reporting five or more persistent symptoms (13 patients). As negative controls and an indicator of normal population, stored plasma samples from 37 individuals who tested negative for SARS-CoV-2 in 2020 were used.

### Metabolomics analysis

A combination of direct injection mass spectrometry with a reverse-phase LC–MS/MS custom assay was used, as previously described^76^. Briefly, metabolites were measured using a locally developed LC–MS/MS metabolomics assay called The Metabolomics Innovation Centre (TMIC) Prime (TMIC PRIME®) Assay. This assay provides quantitative results for up to 143 endogenous metabolites, including biogenic amines, amino acids, organic acids, lipids, and lipid-like compounds.

The method combines the derivatization and extraction of analytes, and the selective mass-spectrometric detection using multiple reaction monitoring (MRM) pairs. Isotope-labeled internal standards and other internal standards were used for metabolite quantification. The custom assay uses a 96 deep-well plate with a filter plate attached via sealing tape, and reagents and solvents used to prepare the plate assay. The first 14 wells of the 96-well plate were used for calibration and quality control with one double blank, three zero samples, seven calibration standards and three quality control samples. To measure all metabolites except organic acids, samples were first thawed on ice and were vortexed. 10 µL of each sample was loaded onto the center of the filter on the upper 96-well plate and dried under a stream of nitrogen. Subsequently, phenyl-isothiocyanate (PITC) was added for derivatization. After incubation, the filter spots were dried again using an evaporator. Extraction of the metabolites was then achieved by adding 300 µL of extraction solvent. The extracts were obtained by centrifugation into the lower 96-deep well plate, followed by a dilution step with the mass spectrometry running solvent.

For organic acid analysis, 150 µL of ice-cold methanol and 10 µL of isotope-labeled internal standard mixture was added to 50 µL of each plasma sample for overnight protein precipitation. Each sample was then centrifuged at 13,000×*g* for 15 min. 50 µL of supernatant was loaded into the center of wells of a 96-deep well plate, followed by the addition of ^13^C labeled 3-nitrophenylhydrazine (3-NPH) as an isotopic labeling reagent (for quantification). After incubation for 2 h, butylated hydroxytoluene (as a stabilizer) and water were added before LC–MS injection.

Mass spectrometric analysis for the PITC-derivatized and 3-NPH-derivatized samples was performed on an ABSciex 4000 Qtrap® tandem mass spectrometry instrument (Applied Biosystems/MDS Analytical Technologies, Foster City, CA) equipped with an Agilent 1260 series UHPLC system. Organic acids, biogenic amines, amino acids, and amino acid derivatives were detected and quantified via LC–MS, while lipids, acylcarnitines, and glucose were detected and quantified via a direct injection (DI) method.

Analyst 1.6.2 and MultiQuant 3.0.3 was used for quantitative analysis. An individual seven-point calibration curve was generated to quantify organic acids, amino acids, biogenic amines, and derivatives. Ratios for each analyte’s signal intensity to its corresponding isotope-labelled internal standard were plotted against the specific known concentrations using quadratic regression with a 1/x^2^ weighting. For lipids, acylcarnitines, and glucose, a single point calibration of a representative analyte was built using the same group of compounds that share the same core structure assuming a linear regression through zero.

### Plasma IL-17 and leptin determinations

ELISA kits were used for the quantification of IL-17 (Catalog Number RAB0262, Sigma-Aldrich, St. Louis, MO, USA) and leptin (catalog number ab108879, Abcam, Cambridge, UK), following manufacturer’s instructions. Briefly, standard solutions (or plasma samples), were added to each type of pre-coated 96-well plate and incubated overnight at 4 °C. The plates were then incubated with the corresponding detection antibodies (100 μL/well) for 1 h at room temperature. Streptavidin solution (100 μL) was then added to each well and the plates were incubated for 45 min. After the antibody-HPR incubation, TMB one-step substrate reagent (100 μL) was added to the wells and the plates were incubated for another 30 min before the addition of a stop solution (50 μL/well). Absorbance values (at 450 nm) were used for the calculation of the protein concentrations (pg/mL) by comparing the absorbance to an appropriate standard curve.

### Statistical analysis

To describe baseline characteristics of negative controls (non-COVID-19), COVID-19 or post-COVID-19 patients, medians with interquartile ranges (IQRs) or means [with standard deviations (s.d.)] and frequencies (%) were used for continuous and categorical data, respectively. Normality was assessed using the D’Agostino-Pearson normality test. Student’s t-test or Mann–Whitney tests were used for continuous data. For categorical variables (e.g., sex, smoking, symptoms, and comorbidities) Pearson Chi^2^ tests or Fisher’s exact tests were used. All p-values less than 0.05 (p < 0.05) were considered statistically significant. Analyses were conducted using SPSS (IBM, version 24).

Metabolite analysis was performed with MetaboAnalyst 5.0^81^. Those metabolites with more than 20% of missing values were removed from further analysis. For the remaining metabolites, values below the limit of detection (LOD) were imputed using 1/5 of the minimum positive value of each variable. The data were then subject to autoscaling to generate appropriate Gaussian metabolite concentration distributions. Differences in mean metabolic values between controls, COVID-19, post-COVID-19, and long COVID patients were assessed using a parametric t-test or one-way ANOVA [adjusted p-value (FDR) cut-off = 0.05]. For the paired study, t-test, and volcano plots of log-transformed p-values were generated to address significant metabolites. Principal component analysis (PCA) and two-dimensional partial least squares discriminant analysis (2-D PLS-DA) scores plots were used to compare plasma metabolite data across and between study groups; 2000-fold permutation tests were used to assess statistical significance and minimize the possibility that the observed separation of the PLS-DA clusters was due to chance. Differentiated metabolites were identified by a variable importance in projection (VIP) using a score cutoff of > 1.5. Heat maps of the top 50 significant metabolites (via t-test or ANOVA) were created via MetaboAnalyst.

Pathway analysis was done using Metabolite Set Enrichment Analysis (MSEA) and Metabolomic Pathway Analysis (MetPA) modules as found in MetaboAnalyst 5.0^81^. The Homo sapiens pathway library was used for pathway analysis. The global test was used for the selected pathway enrichment analysis method, whereas the node importance measure for topological analysis was used to assess the relative betweenness centrality.

The metabolites with the highest VIP scores were used to create metabolite panels for predicting long COVID using multivariate logistic regression. Additionally, models were adjusted for relevant potential confounders such as sex, age, relevant comorbidities (i.e., DM-II, HTN, and obesity), so that only statistically significant variables (p < 0.05) remained in the final models. Logistic regression analysis was performed with the auto-scaled data. K-fold cross-validation (CV) was used to ensure that the logistic regression models were robust. To determine the performance of each generated model, the area under the receiver operating characteristics curve (AUROC or AUC) was calculated, as was sensitivity and specificity.

### Ethics declarations

This study was conducted in accordance with the Declaration of Helsinki (1976). It was also revised and approved by the Research and Ethics Committees of the Instituto Mexicano de Seguridad Social, with the registration number R-2022-3301-038, and Christus Muguerza del Parque Hospital (HCMP-CEI-15042020-3, and HCMP-CEI-28022022-A01). Informed consent was obtained from all participants. All patients included in this study were informed in writing regarding the collection of their samples for research aims and were given the right to refuse participation.

## Supplementary Information

Supplementary Information.

## Data Availability

The datasets generated and analyzed during the current study are available in the Mendeley repository (https://data.mendeley.com) (https://doi.org/10.17632/gc9g2g53kr.1).

## References

[1] Lam W, Zhong N, Tan W (2003). Overview on SARS in Asia and the World. Respirology.

[2] Al Mutair A, Ambani Z (2020). Narrative review of Middle East respiratory syndrome coronavirus (MERS-CoV) infection: Updates and implications for practice. J. Int. Med. Res..

[3] Davis HE, McCorkell L, Vogel JM, Topol EJ (2023). Long COVID: Major findings, mechanisms and recommendations. Nat. Rev. Microbiol..

[4] Chopra V, Flanders SA, O’Malley M, Malani AN, Prescott HC (2021). Sixty-day outcomes among patients hospitalized with COVID-19. Ann. Intern. Med..

[5] Wallace PG (1991). Epidemiology: A critical review. Br. Med. Bull..

[6] Hickie I (2006). Post-infective and chronic fatigue syndromes precipitated by viral and non-viral pathogens: Prospective cohort study. BMJ.

[7] Kukla M (2020). COVID-19, MERS and SARS with concomitant liver injury—systematic review of the existing literature. J. Clin. Med..

[8] Soriano JB, Murthy S, Marshall JC, Relan P, Diaz JV (2022). A clinical case definition of post-COVID-19 condition by a Delphi consensus. Lancet Infect. Dis..

[9] O’Mahoney LL (2023). The prevalence and long-term health effects of Long Covid among hospitalised and non-hospitalised populations: A systematic review and meta-analysis. EClinicalMedicine.

[10] Lopez-Leon S (2021). More than 50 long-term effects of COVID-19: A systematic review and meta-analysis. Sci. Rep..

[11] Wu X (2021). 3-month, 6-month, 9-month, and 12-month respiratory outcomes in patients following COVID-19-related hospitalisation: A prospective study. Lancet Respir. Med..

[12] Schlemmer F (2023). Respiratory recovery trajectories after severe-to-critical COVID-19: A 1-year prospective multicentre study. Eur. Respir. J..

[13] Pintos-Pascual I (2022). Is SARS-CoV-2 the only cause of long-COVID?. AIDS Rev..

[14] Herrera-Van Oostdam AS (2021). Immunometabolic signatures predict risk of progression to sepsis in COVID-19. PLoS ONE.

[15] Chang R (2023). Risk of autoimmune diseases in patients with COVID-19: A retrospective cohort study. EClinicalMedicine.

[16] Almeria M, Cejudo JC, Sotoca J, Deus J, Krupinski J (2020). Cognitive profile following COVID-19 infection: Clinical predictors leading to neuropsychological impairment. Brain Behav. Immun. Health.

[17] Montefusco L (2021). Acute and long-term disruption of glycometabolic control after SARS-CoV-2 infection. Nat. Metab..

[18] Negahdaripour M (2021). Post-COVID-19 hyperglycemia: A concern in selection of therapeutic regimens. Iran J. Med. Sci..

[19] Al-Hakeim HK (2023). Increased insulin resistance due to Long COVID is associated with depressive symptoms and partly predicted by the inflammatory response during acute infection. Braz. J. Psychiatry.

[20] Germain A, Ruppert D, Levine SM, Hanson MR (2017). Metabolic profiling of a myalgic encephalomyelitis/chronic fatigue syndrome discovery cohort reveals disturbances in fatty acid and lipid metabolism. Mol. Biosyst..

[21] Holmes E (2021). Incomplete systemic recovery and metabolic phenoreversion in post-acute-phase nonhospitalized COVID-19 patients: Implications for assessment of post-acute COVID-19 syndrome. J. Proteome Res..

[22] Ticinesi A, Guerra A, Nouvenne A, Meschi T, Maggi S (2023). Disentangling the complexity of nutrition, frailty and gut microbial pathways during aging: A focus on hippuric acid. Nutrients.

[23] Wu G (2011). Proline and hydroxyproline metabolism: Implications for animal and human nutrition. Amino Acids.

[24] Phang JM (2023). The regulatory mechanisms of proline and hydroxyproline metabolism: Recent advances in perspective. Front. Oncol..

[25] Kumar Srivastava A, Khare P, Kumar Nagar H, Raghuwanshi N, Srivastava R (2016). Hydroxyproline: A potential biochemical marker and its role in the pathogenesis of different diseases. Curr. Protein Pept. Sci..

[26] Lange, S. M. & Parekh, M. *Collagen Vascular Disease Associated with Interstitial Lung* (2023). 32644520

[27] Shen B (2020). Proteomic and metabolomic characterization of COVID-19 patient sera. Cell.

[28] Wu P (2021). The trans-omics landscape of COVID-19. Nat. Commun..

[29] Atila A (2021). The serum amino acid profile in COVID-19. Amino Acids.

[30] Masoodi M (2022). Disturbed lipid and amino acid metabolisms in COVID-19 patients. J. Mol. Med..

[31] Ansone L (2021). Amino acid metabolism is significantly altered at the time of admission in hospital for severe COVID-19 patients: Findings from longitudinal targeted metabolomics analysis. Microbiol. Spectr..

[32] Doğan HO (2021). Understanding the pathophysiological changes via untargeted metabolomics in COVID-19 patients. J. Med. Virol..

[33] Liu X (2019). High plasma glutamate and low glutamine-to-glutamate ratio are associated with type 2 diabetes: Case-cohort study within the PREDIMED trial. Nutr. Metab. Cardiovasc. Dis..

[34] Newgard CB (2009). A branched-chain amino acid-related metabolic signature that differentiates obese and lean humans and contributes to insulin resistance. Cell Metab..

[35] Aydın H (2023). Glutamine-driven metabolic adaptation to COVID-19 infection. Indian J. Clin. Biochem..

[36] Yesilkaya UH, Sen M, Balcioglu YH (2021). COVID-19-related cognitive dysfunction may be associated with transient disruption in the DLPFC glutamatergic pathway. J. Clin. Neurosci..

[37] Madeira C (2018). Blood levels of glutamate and glutamine in recent onset and chronic schizophrenia. Front. Psychiatry.

[38] Aldana BI (2020). Glutamate-glutamine homeostasis is perturbed in neurons and astrocytes derived from patient iPSC models of frontotemporal dementia. Mol. Brain.

[39] Guntur VP (2022). Signatures of mitochondrial dysfunction and impaired fatty acid metabolism in plasma of patients with post-acute sequelae of COVID-19 (PASC). Metabolites.

[40] Engel KM, Schiller J, Galuska CE, Fuchs B (2021). Phospholipases and reactive oxygen species derived lipid biomarkers in healthy and diseased humans and animals: A focus on lysophosphatidylcholine. Front. Physiol..

[41] Drobnik W (2003). Plasma ceramide and lysophosphatidylcholine inversely correlate with mortality in sepsis patients. J. Lipid Res..

[42] Che X (2022). Metabolomic evidence for peroxisomal dysfunction in myalgic encephalomyelitis/chronic fatigue syndrome. Int. J. Mol. Sci..

[43] Xu E, Xie Y, Al-Aly Z (2023). Risks and burdens of incident dyslipidaemia in long COVID: A cohort study. Lancet Diabetes Endocrinol..

[44] Albóniga OE (2022). Metabolic snapshot of plasma samples reveals new pathways implicated in SARS-CoV-2 pathogenesis. J. Proteome Res..

[45] Kim HS (2021). Do an altered gut microbiota and an associated leaky gut affect COVID-19 severity?. mBio.

[46] Firpo MR (2021). Targeting polyamines inhibits coronavirus infection by reducing cellular attachment and entry. ACS Infect. Dis..

[47] Reese JT (2023). Generalisable long COVID subtypes: Findings from the NIH N3C and RECOVER programmes. EBioMedicine.

[48] Thaweethai T (2023). Development of a definition of postacute sequelae of SARS-CoV-2 infection. JAMA.

[49] Xu J (2021). Plasma metabolomic profiling of patients recovered from coronavirus disease 2019 (COVID-19) with pulmonary sequelae 3 months after discharge. Clin. Infect. Dis..

[50] Ghali A (2019). Elevated blood lactate in resting conditions correlate with post-exertional malaise severity in patients with Myalgic encephalomyelitis/Chronic fatigue syndrome. Sci. Rep..

[51] Kennedy G (2005). Oxidative stress levels are raised in chronic fatigue syndrome and are associated with clinical symptoms. Free Radic. Biol. Med..

[52] Lane RJM (1998). Muscle fibre characteristics and lactate responses to exercise in chronic fatigue syndrome. J. Neurol. Neurosurg. Psychiatry.

[53] Lane RJM, Barrett MC, Taylor DJ, Kemp GJ, Lodi R (1998). Heterogeneity in chronic fatigue syndrome: Evidence from magnetic resonance spectroscopy of muscle. Neuromuscul. Disord..

[54] Petrović V (2008). Antioxidative defence alterations in skeletal muscle during prolonged acclimation to cold: Role of l-arginine/NO-producing pathway. J. Exp. Biol..

[55] de Boer E (2022). Decreased fatty acid oxidation and altered lactate production during exercise in patients with post-acute COVID-19 syndrome. Am. J. Respir. Crit. Care Med..

[56] Debray F-G (2007). Diagnostic accuracy of blood lactate-to-pyruvate molar ratio in the differential diagnosis of congenital lactic acidosis. Clin. Chem..

[57] Yamano E (2016). Index markers of chronic fatigue syndrome with dysfunction of TCA and urea cycles. Sci. Rep..

[58] Walker V (2012). Severe hyperammonaemia in adults not explained by liver disease. Ann. Clin. Biochem..

[59] Saligan LN, Lukkahatai N, Jin Zhang Z, Cheung CW, Min Wang X (2018). Altered Cd8+ T lymphocyte response triggered by arginase 1: Implication for fatigue intensification during localized radiation therapy in prostate cancer patients. Neuropsychiatry.

[60] Ryan FJ (2022). Long-term perturbation of the peripheral immune system months after SARS-CoV-2 infection. BMC Med..

[61] Nuber-Champier A (2022). Monocytosis in the acute phase of SARS-CoV-2 infection predicts the presence of anosognosia for cognitive deficits in the chronic phase. Brain Behav. Immun. Health.

[62] McGeachy MJ, Cua DJ, Gaffen SL (2019). The IL-17 family of cytokines in health and disease. Immunity.

[63] Garmendia JV, García AH, De Sanctis CV, Hajdúch M, De Sanctis JB (2022). Autoimmunity and immunodeficiency in severe SARS-CoV-2 infection and prolonged COVID-19. Curr. Issues Mol. Biol..

[64] Lou F (2020). Excessive polyamine generation in keratinocytes promotes self-RNA sensing by dendritic cells in psoriasis. Immunity.

[65] Bazdyrev E (2021). Lung fibrosis after COVID-19: Treatment prospects. Pharmaceuticals.

[66] Saini L (2022). Post-COVID-19 immune-mediated neurological complications in children: An ambispective study. Pediatr. Neurol..

[67] Montoya JG (2017). Cytokine signature associated with disease severity in chronic fatigue syndrome patients. Proc. Natl. Acad. Sci. USA.

[68] Procaccini C, Pucino V, Mantzoros CS, Matarese G (2015). Leptin in autoimmune diseases. Metabolism.

[69] Stringer EA (2013). Daily cytokine fluctuations, driven by leptin, are associated with fatigue severity in chronic fatigue syndrome: Evidence of inflammatory pathology. J. Transl. Med..

[70] Tosato M (2022). Effects of l-arginine plus vitamin C supplementation on physical performance, endothelial function, and persistent fatigue in adults with long COVID: A single-blind randomized controlled trial. Nutrients.

[71] Kirby T (2021). COVID-19 survivor experiencing long-term symptoms. Lancet Respir. Med..

[72] Girón Pérez DA (2022). Post-COVID-19 syndrome in outpatients and its association with viral load. Int. J. Environ. Res. Public Health.

[73] Davis HE, McCorkell L, Vogel JM, Topol EJ (2023). Long COVID: Major findings, mechanisms and recommendations. Nat. Rev. Microbiol..

[74] Pfaff ER (2023). Coding long COVID: Characterizing a new disease through an ICD-10 lens. BMC Med..

[75] Ballering AV, van Zon SKR, olde Hartman TC, Rosmalen JGM (2022). Persistence of somatic symptoms after COVID-19 in the Netherlands: An observational cohort study. The Lancet.

[76] López-Hernández Y (2021). Targeted metabolomics identifies high performing diagnostic and prognostic biomarkers for COVID-19. Sci. Rep..

[77] Shear MK (2001). Reliability and validity of a structured interview guide for the Hamilton Anxiety Rating Scale (SIGH-A). Depress Anxiety.

[78] Hamilton M (1960). A rating scale for depression. J. Neurol. Neurosurg. Psychiatry.

[79] Nasreddine ZS (2005). The Montreal Cognitive Assessment, MoCA: A brief screening tool for mild cognitive impairment. J. Am. Geriatr. Soc..

[80] Mahler DA, Wells CK (1988). Evaluation of clinical methods for rating dyspnea. Chest.

[81] Xia J, Psychogios N, Young N, Wishart DS (2009). MetaboAnalyst: A web server for metabolomic data analysis and interpretation. Nucleic Acids Res..

