# Supplementary material for: The plasma metabolome of long COVID patients two years after infection
Source: Sci Rep. 2023 Aug 1;13:12420. doi: 10.1038/s41598-023-39049-x (PMC10394026; doi:10.1038/s41598-023-39049-x)
Supplement: Supplementary file 1 — Supplementary Information. [file 41598_2023_39049_MOESM1_ESM.docx]

**The plasma metabolome of long COVID patients two years after infection**

Yamilé López-Hernández^1*^, Joel Monárrez-Espino^2^, David Alejandro García López^3^, Jiamin Zheng^4^, Juan Carlos Borrego^5^, Claudia Torres-Calzada^6^, José Pedro Elizalde-Díaz^7^, Rupasri Mandal^4^, Mark Berjanskii^4^, Eduardo Martínez-Martínez^7^, Jesús Adrián López^8^, David S. Wishart^4,6^*

^1^CONACyT- Metabolomics and Proteomics Laboratory, Academic Unit of Biological Sciences, Autonomous University of Zacatecas, Zacatecas, 98000, Mexico.

^2^Department of Health Research. Christus Muguerza del Parque Hospital – University of Monterrey. Chihuahua, 31125, Mexico.

^3^Academic Unit of Biological Sciences, Autonomous University of Zacatecas, Zacatecas, 98000, Mexico.

^4^The Metabolomics Innovation Centre, University of Alberta, Edmonton, AB T6G 1C9, Canada.

^5^Departamento de Epidemiología, Hospital General de Zona #1 “Emilio Varela Luján”, Instituto Mexicano del Seguro Social, Zacatecas, 98000, México.

^6^Department of Biological Sciences, University of Alberta, Edmonton, AB T6G 1C9, Canada

^7^Laboratory of Cell Communication & Extracellular Vesicles, Division of Basic Science, Instituto Nacional de Medicina Genómica, Ciudad de México, 14610, Mexico.

^8^MicroRNAs and Cancer Laboratory, Academic Unit of Biological Sciences, Autonomous University of Zacatecas, Zacatecas, 98000, Mexico.

**Corresponding authors**

***** [dwishart@ualberta.ca](mailto:dwishart@ualberta.ca); [ylopezher@conacyt.mx](mailto:ylopezher@conacyt.mx)

**Supplementary Figures**


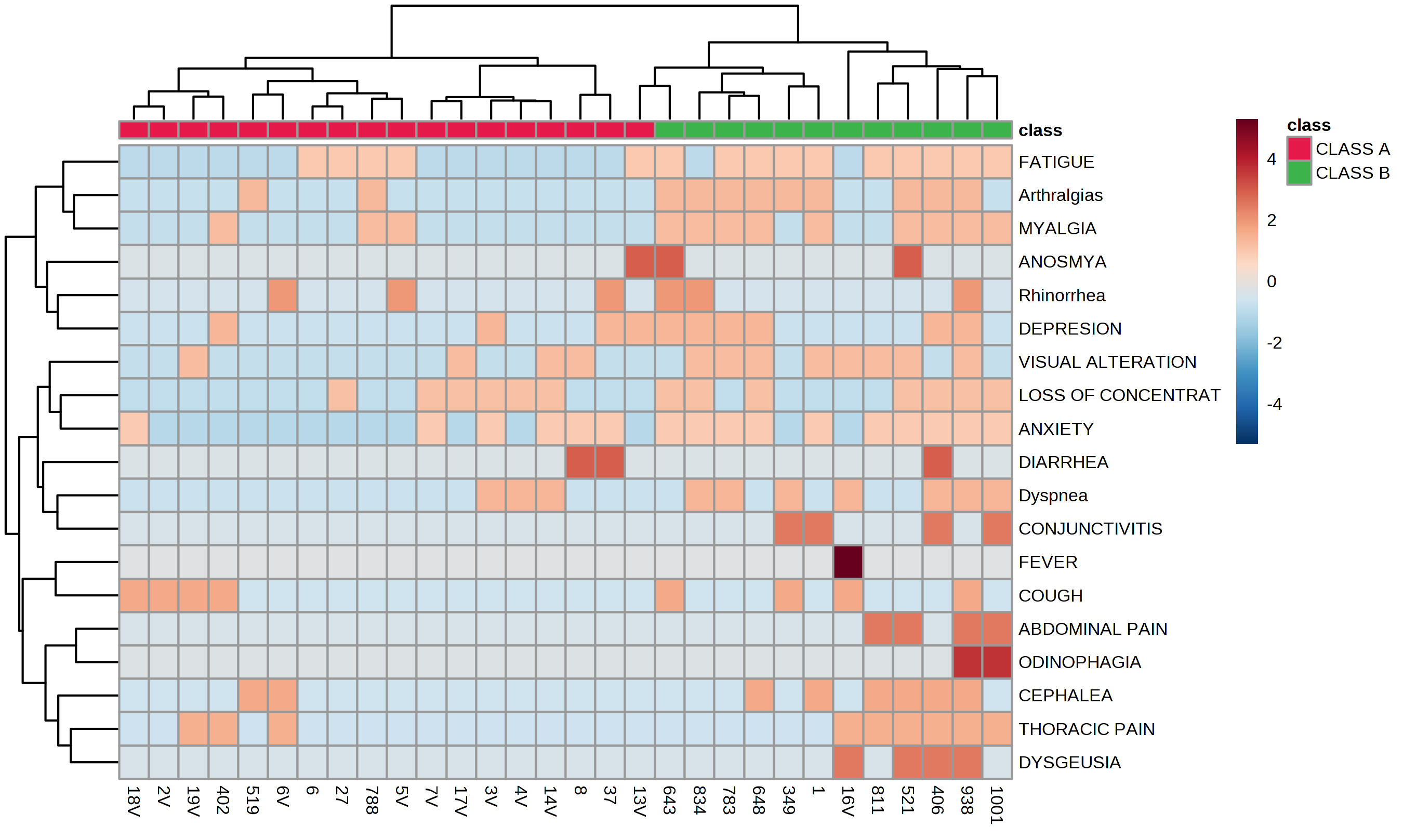


**Supplementary Figure 1:** Heat map representing number and distribution of symptoms among Class A and Class B patients.


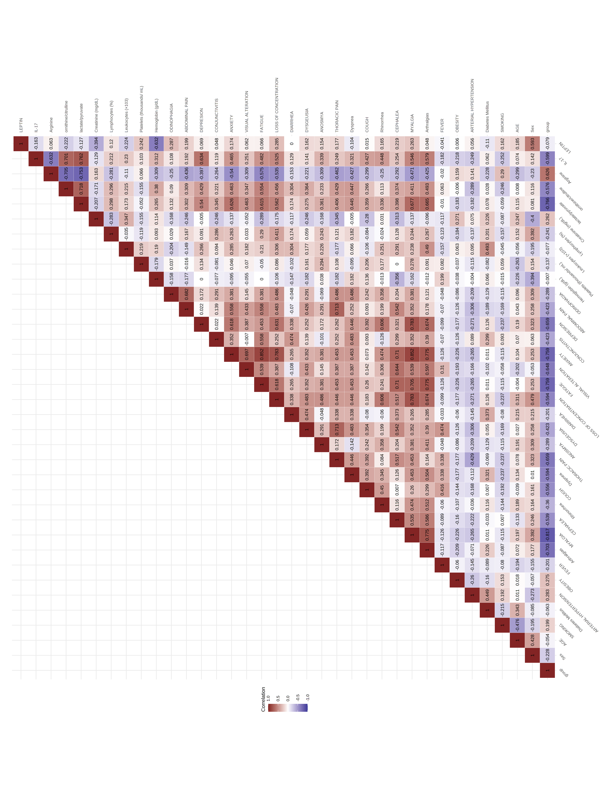


**Supplementary Figure 2:** Correlation matrix. The figure shows a map of the Spearman correlation between the clinical data and significant immunometabolic variables. Red boxes presented a positive correlation, purple boxes presented a negative correlation. blue Darker colors represent stronger correlation.

**Supplementary Figure 3:** Representative Computerized tomography scans (CT scans) from three Class B patients (2020 and 2022). 65-year-old female patient. (a) Initial evaluation (2020) showing lesions with ground glass pattern and baseline images of consolidation. (b) Follow-up (2022) showing predominantly medial and basal involvement with areas of right parahilar infiltration and persistent basal fibrosis predominantly basal. 67-year-old female patient. (c) Initial evaluation (2020) showing a consolidation pattern with areas of right parahilar infiltration. (d) Follow-up (2022) showing the presence of scattered infiltrates in the middle and basal right and left levels with a fibrotic pattern. 42-year-old female patient. (e) Initial evaluation (2020) showing medial affection with areas of bilateral consolidations at the basal region. (d) Follow-up (2022) showing the presence of minimal infiltrates at medium levels with a fibrotic pattern.

**Supplementary Tables**

Supplementary Table 1: Clinical characteristics of class B, class A long COVID patients and recovered (non-long COVID).

|  | **CLASS B** | **CLASS A** | **RECOVERED** |
| --- | --- | --- | --- |
| Sex(Male), n(%) | 6(50) | 7(36.8) | 6(33.3) |
| Smoking n(%) | 1(8.3) | 2(10.5) | 4(22.2) |
| DM n(%) | 3(25) | 1(5.3) | 3(16.7) |
| Hypertension n(%) | 3(25) | 9(47.4) | 9(50) |
| Obesity n(%) | 0(0) | 2(10.5) | 3(16.7) |
| Hb (g/dL, Mean+/-SD) | 15.1+/-2 | 15.1+/-1.8 | 18+/-1.7 |
| Platelets (thousands/ mL, Mean+/-SD) | 241+/-50 | 255+/-80 | 247+/-43.9 |
| Leukocytes (×10^3^, Mean+/-SD) | 8.9+/-1.9 | 6.53+/-0.9 | 7.5+/-1.9 |
| Lymphocytes (%, Mean+/-SD) | 35+/-5.6 | 34+/-8.5 | 30.7+/-6.9 |
| Creatinine (mg/dL, Mean+/-SD) | 0.8+/-0.27 | 0.8+/-0.1 | 0.9+/-0.3 |
